# Autotaxin-Lysophosphatidic Acid Axis Is a Novel Molecular Target for Lowering Intraocular Pressure

**DOI:** 10.1371/journal.pone.0042627

**Published:** 2012-08-20

**Authors:** Padma Iyer, Robert Lalane, Corey Morris, Pratap Challa, Robin Vann, Ponugoti Vasantha Rao

**Affiliations:** 1 Department of Ophthalmology, Duke University School of Medicine, Durham, North Carolina, United States of America; 2 Department of Pharmacology and Cancer Biology, Duke University School of Medicine, Durham, North Carolina, United States of America; Aligarh Muslim University, India

## Abstract

Primary open-angle glaucoma is the second leading cause of blindness in the United States and is commonly associated with elevated intraocular pressure (IOP) resulting from diminished aqueous humor (AH) drainage through the trabecular pathway. Developing effective therapies for increased IOP in glaucoma patients requires identification and characterization of molecular mechanisms that regulate IOP and AH outflow. This study describes the identification and role of autotaxin (ATX), a secretory protein and a major source for extracellular lysophosphatidic acid (LPA), in regulation of IOP in a rabbit model. Quantitative proteomics analysis identified ATX as an abundant protein in both human AH derived from non-glaucoma subjects and in AH from different animal species. The lysophospholipase D (LysoPLD) activity of ATX was found to be significantly elevated (by ∼1.8 fold; n = 20) in AH derived from human primary open angle glaucoma patients as compared to AH derived from age-matched cataract control patients. Immunoblotting analysis of conditioned media derived from primary cultures of human trabecular meshwork (HTM) cells has confirmed secretion of ATX and the ability of cyclic mechanical stretch of TM cells to increase the levels of secreted ATX. Topical application of a small molecular chemical inhibitor of ATX (S32826), which inhibited AH LysoPLD activity *in vitro* (by >90%), led to a dose-dependent and significant decrease of IOP in Dutch-Belted rabbits. Single intracameral injection of S32826 (∼2 µM) led to significant reduction of IOP in rabbits, with the ocular hypotensive response lasting for more than 48 hrs. Suppression of ATX expression in HTM cells using small-interfering RNA (siRNA) caused a decrease in actin stress fibers and myosin light chain phosphorylation. Collectively, these observations indicate that the ATX-LPA axis represents a potential therapeutic target for lowering IOP in glaucoma patients.

## Introduction

Glaucoma, a leading cause of blindness characterized by optic nerve degeneration and progressive visual field loss, is commonly associated with elevated intraocular pressure (IOP) [1]. Ocular hypertension (OH) or elevated IOP is a definite and primary risk factor for primary open angle glaucoma (POAG) [1], [2], [3]. The risk of developing glaucoma decreases significantly when a 20% reduction in IOP is achieved in patients with OH, and ocular hypotensive therapy remains the mainstay of glaucoma treatment [4], [5]. However, while there are several classes of ocular hypotensive drugs available for treatment of glaucoma, there remains a significant unmet medical need for novel, more efficacious and targeted therapy [5], [6]. This need requires that we address the gap which currently exists in our understanding of regulation of IOP and AH outflow and identification of physiological and pathological factors that influence IOP and AH outflow in both normal and glaucoma patients.

The conventional or trabecular AH outflow pathway is composed of the trabecular meshwork (TM), juxtacanalicular connective tissue (JCT) and Schlemm's canal (SC). In humans, this pathway represents a predominant route of AH drainage [3]. AH is secreted by non-pigmented epithelial cells that line the ciliary body and flows into the anterior chamber, which then drains through the TM into Schlemm's canal and the episcleral veins on a continuous basis. While there is a general agreement that decreased AH outflow through the trabecular pathway is the primary cause for increased IOP in glaucoma patients, little is known about the molecular basis for increased resistance to AH outflow through the trabecular pathway [3], [7]. Various extracellular factors including TGF-beta, bioactive lipids (lysophosphatidic acid and Sphingosine-1 phosphate), endothelin-1, myocilin, interleukins, prostaglandins, steroids, CD44, matrix metalloproteinases and extracellular matrix proteins have been reported to influence AH outflow and IOP [3], [8], [9], [10], [11], [12], [13], [14], [15], [16], [17], [18]. Importantly, the levels of some of these factors have been shown to be elevated in the AH of human glaucoma patients [2], [13], [19], [20], [21].

In our previous work, we demonstrated that TM and SC cells express different G-protein coupled receptors specific for both lysophosphatidic acid (LPA) and sphingosin-1-phosphate (S1P), and perfusion of enucleated eyes with these lipids was noted to influence AH outflow concomitant with changes in TM cell contractile properties, Rho GTPase activation and expression of extracellular matrix proteins [15], [22]. These initial observations were subsequently confirmed by independent investigators and collectively supported the importance of bioactive lipids in regulation of AH outflow and potentially IOP [15], [17], [23]. Although these observations implied the significance of LPA and SIP in the regulation of AH outflow, we currently know very little about how the levels of these bioactive lipids are regulated in AH, and the different molecular mechanisms involved in the generation of these lipids in the AH. To explore these questions, we recently performed quantitative proteomic analysis of human AH derived from cataract surgery patients with normal IOP. This initial analysis revealed that ATX [24], which is a well characterized secretory protein with lysophospholipase D (LysoPLD) activity, was one of the abundant proteins in human AH. Since this protein has been considered a major source for extracellular LPA [25], [26], and LPA has in turn been demonstrated to influence AH outflow facility [15], in this study we investigated the role of ATX in IOP using a small molecular weight chemical inhibitor of this protein. This study reports that inhibition of ATX LysoPLD activity by topical and intracameral delivery of a chemical inhibitor significantly decreases IOP, suggesting that ATX is a potential therapeutic target for lowering IOP in glaucoma patients.

## Results

### ATX is an abundant protein of human aqueous humor

Towards our objective to identifying extracellular factors regulating AH outflow and IOP, an LC-MS (liquid chromatography- mass spectrometry) proteomic analysis was performed to identify abundant proteins of human AH, using one µg of pooled AH total protein derived from 8 individual donors who underwent cataract surgery. This analysis identified eighty nine non-redundant proteins with high confidence based on 2 or more matching peptides. Individual proteins were quantified by comparison of signal intensity, using an internal standard of yeast alcohol dehydrogenase (label free). Table S1 lists AH proteins identified by proteomic analysis, together with their respective Swiss-Prot protein accession number, protein common name and relative concentration (fmol/1 µg total protein). Of the different easily detectable proteins present in the human AH sample, ATX, also known as Ectonucleotide pyrophosphatase/phosphodiesterase (ENNP2), was found to be one of 25 most abundant proteins (Table S1).

**Figure 1:**
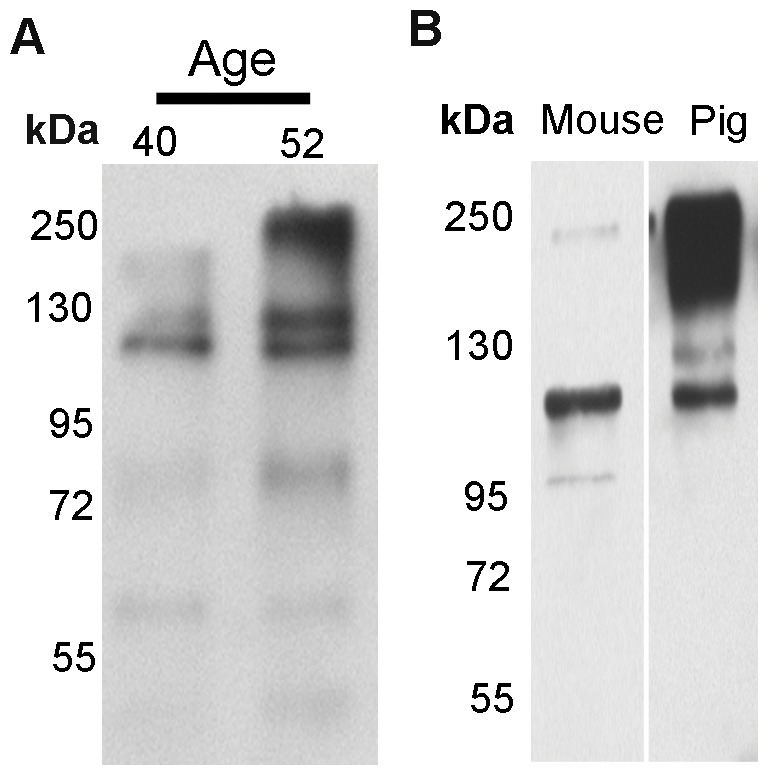
Detection of ATX in the AH from human and other species. **A**. Immunoblot analysis of AH (10 µg protein) derived from two different human donors (aged 40 and 52 years) revealed multiple immunopositive bands with molecular mass ranging between 65 to 250 kDa. B. Detection of ATX by immunoblot analysis of mouse and porcine AH (10 µg protein). Similar to human AH, both mouse and porcine exhibit ATX immunopositive bands with molecular mass ranging from 95 to 250 kDa.

To independently confirm the presence of ATX in AH by an alternative means, we performed immunoblotting analysis of AH using a rabbit polyclonal anti-ATX antibody. Immunoblotting analysis of 10 µg of human AH total protein derived from two independent donors (aged 40 and 52 years) identified multiple immunopositive bands with molecular mass ranging from 65 to 250 kDa, confirming the presence of ATX isoforms in different proteolytically and postranslationally processed forms (Fig. 1A). Protein blots of the AH sample were also probed with primary antibody which was pre-blocked with antigen specific peptide or secondary antibody alone, to confirm specificity of the primary antibody used for immunoblotting, and confirmed lack of non-specific reactions in the observed results. We also confirmed the presence of ATX in mouse and porcine AH by immunoblot analysis (Fig. 1B) and comparative analysis revealed interesting differences and similarities in the profiles of ATX isoforms between the human, mouse and porcine species.

### Elevated levels of LysoPLD activity in the AH of POAG patients

**Figure 2:**
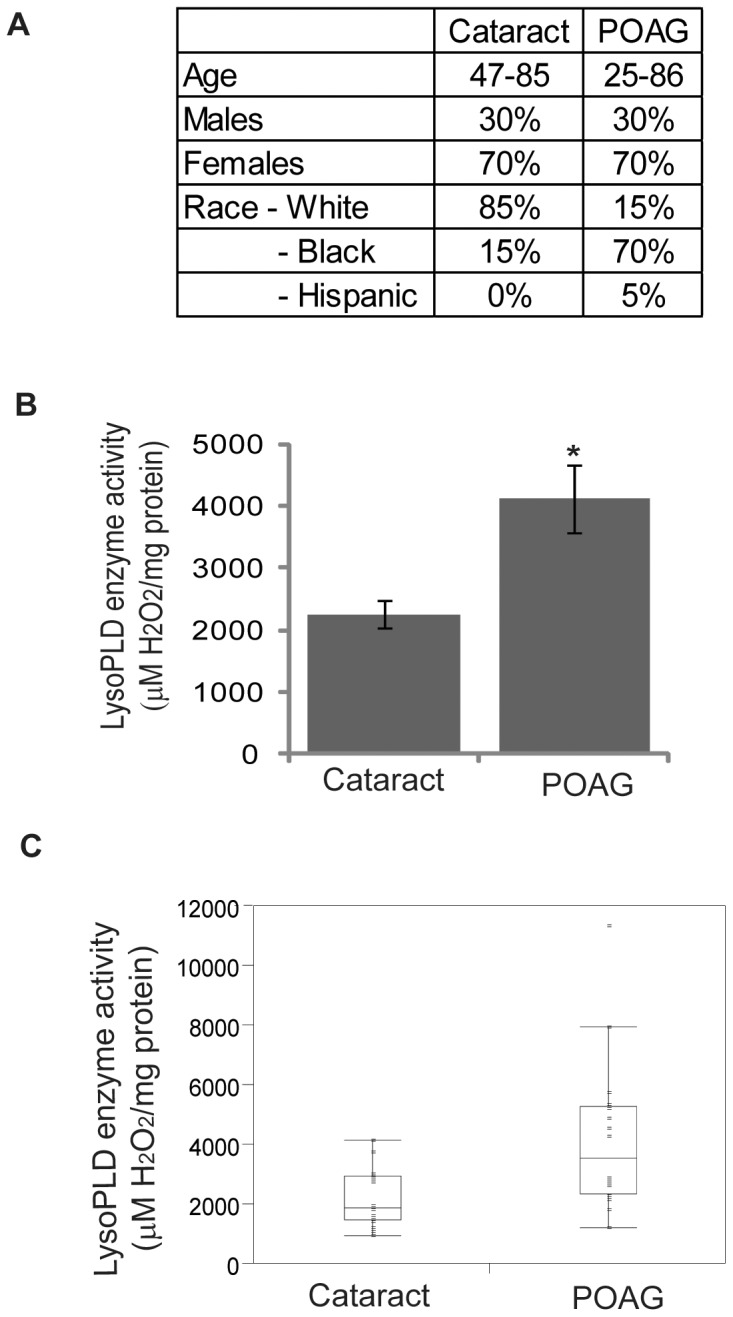
Elevated levels of LysoPLD activity in AH derived from primary open angle glaucoma human patients. A. Details of the demographics for the Cataract control and POAG patients. B. LysoPLD activity (expressed per unit of AH total protein) in the AH of POAG patients (n = 20) revealed a significant (* p< = 0.005) increase relative to age-matched cataract control specimens based on the Wilcoxon Rank Sum test. C. Box-and-whisker plot of the AH LysoPLD activity of glaucoma and cataract controls with box showing median and interquartile range.

To measure LysoPLD activity in human AH, we utilized a modified fluorogenic enzyme-coupled assay protocol [27] using a commercially available Amplex Red PLD Assay kit. Human AH samples derived from 20 different cataract and POAG patients were tested for LysoPLD activity. While age and sex were matched between the cataract and glaucoma groups, race demography was found to be significantly (p<0.05) different between the two groups based on both Fisher's exact and Chi-Square tests. As shown in Figure 2A, the racial composition of the POAG sample group consisted of 75% African American, 15% Caucasian/white and 5% Hispanic patients. On the other hand, the cataract control group comprised of 85% Caucasian/white and 15% African American patients. LysoPLD activity measured in these specimens was expressed as micromoles of H_2_O_2_ generated per milligram of AH protein, with the activity measured for POAG patients being significantly elevated (by 1.8 fold, n = 20), as compared to the cataract control specimen values, based on the Wilcoxon Rank Sum test of differences in medians with one-way Chi-Square approximation (Fig. 2B and 2C). Figure 2C provides Box-and-whisker plots of the AH LysoPLD activity of glaucoma and cataract controls, with the box showing median and interquartile ranges.

### ATX is expressed and secreted by TM cells

**Figure 3:**
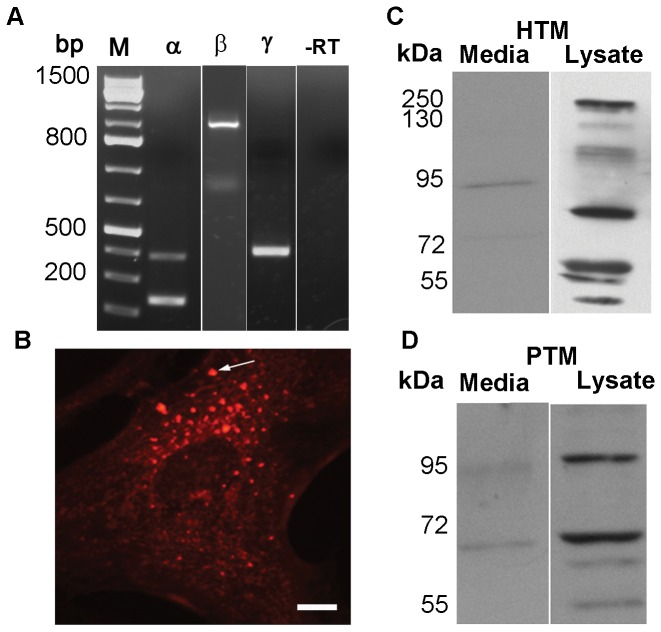
Expression and secretion of ATX by primary cultures of trabecular meshwork cells derived from human and porcine eyes. A. RT-PCR based amplification of total RNA derived from human TM cells confirmed expression of the α, β and γ variants of ATX. PCR products were sequenced to confirm identity. Similar observations were noted in TM cells from three different human donors. -RT represents negative controls run without reverse transcriptase. B. Immunofluorescence localization of ATX in primary cultures of human TM cells reveals a vesicular/punctate distribution, characteristic of a secretory protein. C and D. Human (C) and porcine (D) TM cell lysates (20 µg protein) and conditioned media from TM cells (10 µg protein) show the presence of various isoforms of ATX by immunoblot analysis. Bar: 10 µm.

Since ATX was easily detectable in human AH samples, we hypothesized that this protein is expressed and secreted by cells of the AH outflow pathway. RT-PCR analysis confirmed expression of all three isoforms of ATX (α, β and γ, Fig. 3A) [28] in primary cultures of HTM cells derived from three different donors (aged 33, 54 and 75 years; representative data from a single TM cell strain (donor) is shown). Examination of the cellular distribution profiles of ATX in primary cultures of HTM cells by immunofluorescence analysis using a polyclonal antibody specific for ATX (Fig. 3B) reveals a distinct vesicular/punctate distribution pattern, which is a characteristic feature of secretory proteins. Immunoblotting analysis of ATX protein profiles in cell lysates (20 µg protein) derived from primary cultures of HTM and porcine TM cells (Fig. 3C and 3D, respectively) revealed multiple immunopositive bands with molecular mass ranging from 55 to 250 kDa. On the other hand, the HTM cell conditioned media (10 µg protein) exhibited a single immunopositive band with a molecular mass of ∼95 kDa (Fig. 3C). Compared to the HTM cells, the PTM cell conditioned media (10 µg) showed the 95 kDa protein similar to the HTM cells and an additional immunopositive band at around∼65 kDa (Fig. 3D).

### In vitro inhibition of AH ATX activity by S32826

**Figure 4:**
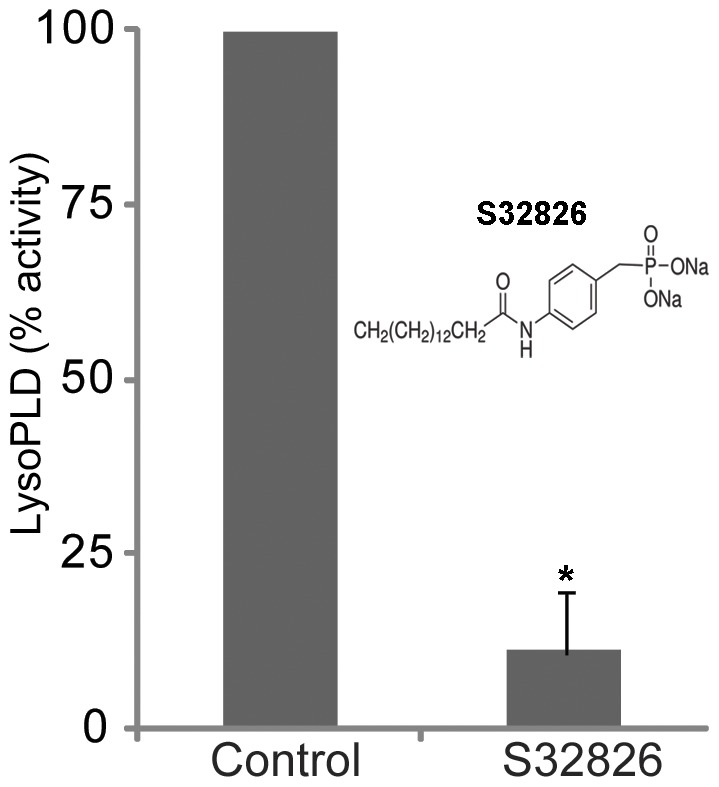
*In vitro* inhibition of rabbit AH LysoPLD activity by S32826. To confirm that S32826 inhibits LysoPLD activity of AH, 10 µg of AH protein derived from rabbits was pre-treated with S32826 *in vitro* for 20 minutes prior to analysis of LysoPLD activity using Amplex Red reagent and fluorimetric detection as described in methods. Rabbit AH LysoPLD activity was inhibited by more than 90% by 10 µM S32826 based on 4 independent observations. The chemical structure of S32826 is shown in the figure inset [29].

For this experiment, the water soluble chemical inhibitor- S32826 [4-(Tetradecanoylamino) benzyl phosphonic acid disodium salt] which inhibits ATX in the nanomolar range [29], was used to assess inhibition of LysoPLD activity in AH derived from the rabbits *in vitro*. Ten microgram aliquots of rabbit AH total protein were incubated with different concentrations (5–25 µM) of S32826 at 37°C for 20 minutes prior to determining LysoPLD activity using Amplex Red reagent [27]. While S32826 is reported to have an IC_50_ in the nanomolar range (9 nM) for purified ATX, and a low IC_50_ for the phosphodiesterase activity of ATX, the IC_50_ for inhibition of LPA release from adipocytes is much higher (200 nM) [29]. Therefore, we used µM range drug concentrations to assess the effect of S32828 in AH samples to account for possible matrix effects of a heterogeneous biological sample. Incubation of rabbit AH with 10 µM S32826 inhibited LysoPLD enzyme activity by 90% (n = 4; p<0.01; based on Student's t-test, Fig. 4).

### Effects of topically administered S32826 on IOP in rabbits

**Figure 5:**
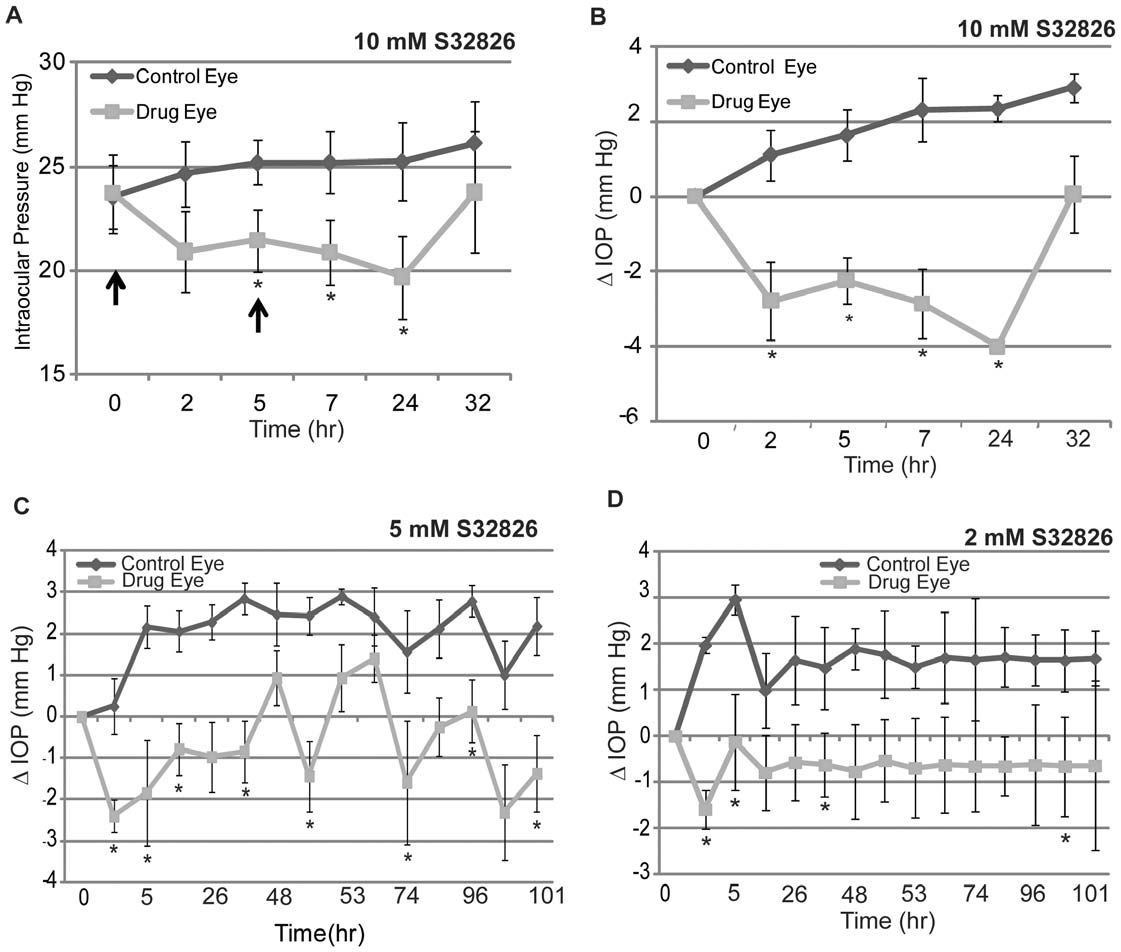
Topical application of ATX inhibitor (S32826) decreases IOP in rabbits. Topical application of S32826 (dissolved in water, pH∼8–9) decreased IOP in a dose- and time- dependent manner in Dutch-Belted adult male rabbits, as determined using pneumotonometer under waking conditions. A. 10 mM S32826 exhibited the maximum and statistically significant effect in lowering IOP (by 5–6 mm Hg; n = 9) with drug application at 0 and 5 hrs (indicated with arrows, 35 µl×2) relative to sham controls receiving pH adjusted water. The IOP-lowering effect of drug lasted for a minimum of 24 hrs, after which, the changes in IOP began to normalize and were similar to control values by 48 hrs after initial drug application. B. The effects of S32826 (10 mM) on net change in IOP from basal values in the same animals. Net IOP values were significantly reduced from basal values, and between 2 to 24 hours following drug administration, based on a paired t-test. Further, IOP in the drug treated eyes was significantly reduced as compared to IOP in the control eyes based on the Wilcoxon Rank Sum test of differences in median's (n = 9). C and D. Effects of 5 and 2 mM S32826 on IOP with a daily (single morning dose, 35 µl×2; n = 4) dose of drug application, respectively. Relative to the 2 mM concentration of drug, administration of 5 mM drug resulted in a significant drop in IOP starting from 2 hrs post drug injection as compared the control values, with the IOP lowering effect lasting till the end of the study (5 days later). While eyes treated with 2 mM S32826 exhibited IOP values that were significantly reduced relative to control values throughout the 5-day study period, based on the Wilcoxon Rank Sum test of differences in medians, significance was not consistent across all time points as shown in the figure. In all these experiments, sham treatment alone increased IOP; the difference was not significant relative to the basal IOP values in any of the experiments. *P<0.05.

**Figure 6:**
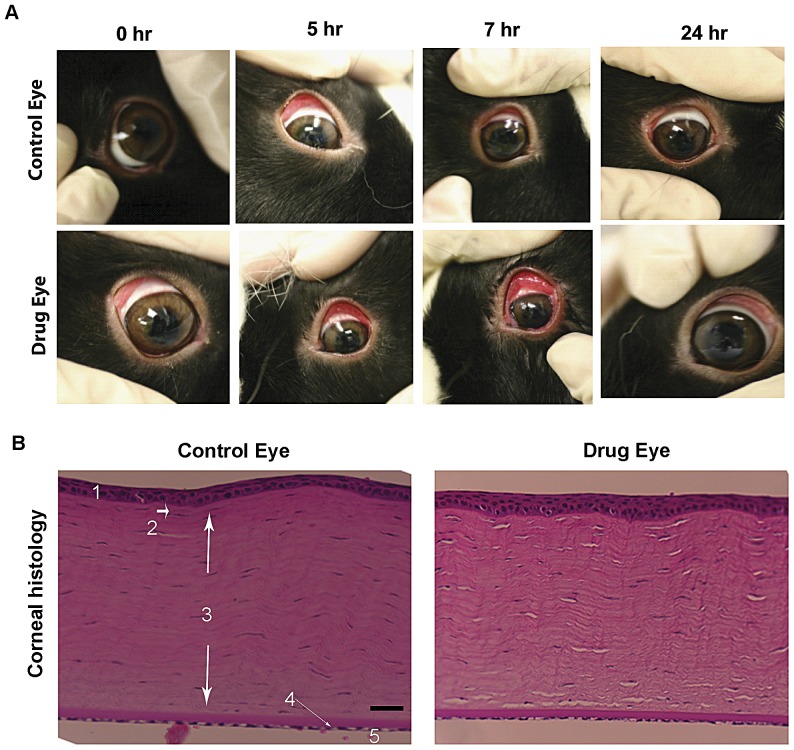
Effects of topical application of S32826 on the rabbit cornea. A. Topical application of 10 mM S32826 (twice daily, 35 µl aliquots) in Dutch-Belted adult male rabbits caused conjunctival hyperemia within the first 2 hrs after drug application, in association with drug precipitation on the cornea and conjunctival tissue as a fine thin film. However, hyperemia and all evidence of drug precipitation disappeared within 24 hrs following drug application. B. Topical effects of S32826 on rabbit corneal histology. Rabbits treated with a topical dose of 5 mM S32826 for 5 days (single daily dose) and which showed a decrease in IOP were enucleated and examined for corneal histological changes relative to sham treated specimens. As shown in the figure, drug application did not cause any apparent change on the integrity of corneal epithelium, endothelium, Bowman's layer, Stroma or Descemet's membrane (based on hematoxylin and eosin staining) relative to the control cornea. Bar: 10 µm. Labels: 1. Corneal Epithelium; 2. Bowman's layer; 3. Corneal Stroma; 4. Descemet's membrane; 5. Corneal Endothelium.

Nine Dutch-Belted normal male rabbits were used in two different experiments (4 and 5 in the first and second experiment, respectively). Prior to drug treatment, IOP was monitored for several days using a pneumotonometer to confirm steady baseline values. Of the three different concentrations (2, 5, and 10 mM) of drug tested topically, the highest dose (10 mM) caused a significant drop in IOP starting 2 hrs and continuing for at least for 24 hrs post-application in all nine rabbits evaluated. IOP values decreased by 10–17% from basal IOPs in the drug treated eyes, and by 15–22% between drug-treated and control eyes (p<0.005, n = 9). Further, this response to drug treatment was consistently observed in three different experiments (twice with the first set of rabbits, and for the third time with a second set of rabbits). Figure 5A shows IOP changes with time, and 5B depicts the net change in IOP values in drug-treated eyes as compared to control eyes. We noted a steady increase or shift in basal IOPs to higher levels in these experiments, although the differences were not significant from the basal value in the control eyes, perhaps due to alkaline sham treatment. Daily topical administration of a 5 mM dose (mornings only) for 5 days also caused a significant, but smaller net decrease in IOP (by ∼3 mm Hg between drug and control groups; n = 4) as compared to sham treated controls (Fig. 5C). In contrast, while topical treatment with the 2 mM (daily morning dose for 5 days) concentration of drug did not yield a steady significant drop in IOP between drug treated and control eyes, IOP values in the drug treated rabbits were consistently lower than in control animals by 1.5 to 2 mm Hg (Fig. 5D). Throughout these experiments, topical application of all three doses of drug dissolved in aqueous medium was associated with drug precipitation on the corneal surface as a fine thin film. Further, topical application of the 10 mM dose of drug was observed to cause conjunctival hyperaemia within the first 2 hrs of drug application, which disappeared within 24 hrs, at which time we still noted a significant decrease in IOP in the drug treated animals compared to the controls (Fig. 6A). Based on slit lamp microscopic examination performed on these animals by a board certified glaucoma specialist, drug treated eyes did not exhibit any noticeable inflammation. Further, post-mortem histology analysis of the cornea (Fig. 6B) and iridocorneal angle (not shown) of rabbit eyes treated with 5 mM drug for 5 days revealed no obvious or toxicological changes relative to control eyes.

### Effects of intracameral administration of S32826 on IOP of rabbits

**Figure 7:**
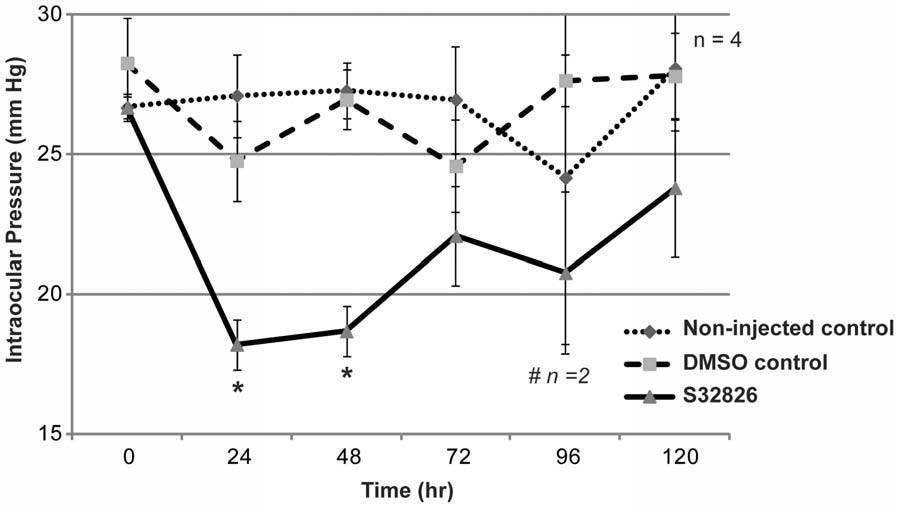
Intracameral injections of ATX inhibitor decreases IOP in rabbits. To determine the effects of intracameral delivery of S32826 on IOP, Dutch belted adult male rabbits (one eye from each animal, n = 4) were intracamerally injected with 5 µl of 100 µM drug. IOP was monitored in drug treated and contralateral (untreated) control eyes using a pneumotonometer. Drug treated eyes showed a significant decrease in IOP from the baseline (by 6–7 mm Hg) at 24 and 48 hrs based on paired t-test. This IOP lowering effect continued for more than 96 hrs, with IOP gradually returning to basal value by120 hrs post- injection. Further, IOP in drug treated eyes was significantly reduced as compared to IOP of the contralateral untreated eyes and eyes treated with sham control (PBS containing 2%DMSO) based on the Wilcoxon Rank Sum test of differences in medians. For the sham treatment, we used the contralateral eyes of the same animals in which we tested the effects of drug, with the sham treatment being performed 2 weeks after completion of the drug treatments (n = 4 for drug treated, sham treated and untreated controls). However, at the 96 hr time point, IOP was recorded in only two of the four rabbits from the drug treated and untreated control groups. *P<0.05.

Since topical drug application was observed to result in drug precipitating on the cornea and causing conjunctival hyperaemia, we decided to change the route of administration. The effects of intracameral injections (delivering the drug directly into the anterior chamber of the eye) were assessed by administering S32826 dissolved in phosphate buffered saline (pH 7.4) at a much lower concentration (µM) than the topical dose (mM). Four Dutch-Belted normal rabbits were used for this experiment. Drug was dissolved initially in dimethyl sulfoxide (DMSO) and subsequently diluted with PBS containing 2% DMSO. In each rabbit one eye was injected with 5 µl of 100 µM drug dissolved in PBS with 2% DMSO, while the other eye served as a non-injected control. The effect of vehicle alone was tested by administering 5 µl of PBS containing 2% DMSO into the contralateral eyes of the same rabbits used for evaluating the effect of active drug. Vehicle administration was performed after waiting at least for one week following completion of the drug treatment experiments, and after ensuring that IOP values were confirmed to be similar to basal values. Intracameral delivery of drug did not result in precipitation as evaluated using a slit lamp microscope. Drug-injected eyes exhibited a significant decrease in IOP (33% (p<0.0036) at 24 hrs, 31.5% (p<0.001) at 48 hrs and 18% (p<0.001) at 72 hrs post drug injection) compared to contralateral non-injected eyes (Fig. 7). Similarly, in comparison to the vehicle injected control eyes, IOP was observed to decrease by 18% (p<0.007) at 24 hrs and 30% (p<0.007) at 48 hrs after drug injection in S32826 treated eyes. Further, IOP values of drug treated eyes were significantly lower relative to basal IOP values (prior to drug injection) measured in the same animals (Fig. 7). At the 96 hr time point post drug injection, we missed recording IOP in two of the animals used in the experiment, resulting in an n = 2 observations for this time point, as indicated in Figure 7. There were no notable ocular abnormalities in any group of animals used in this experiment, either during or after completion of the tests described.

### Effects of siRNA-based suppression of ATX expression in TM cells

**Figure 8:**
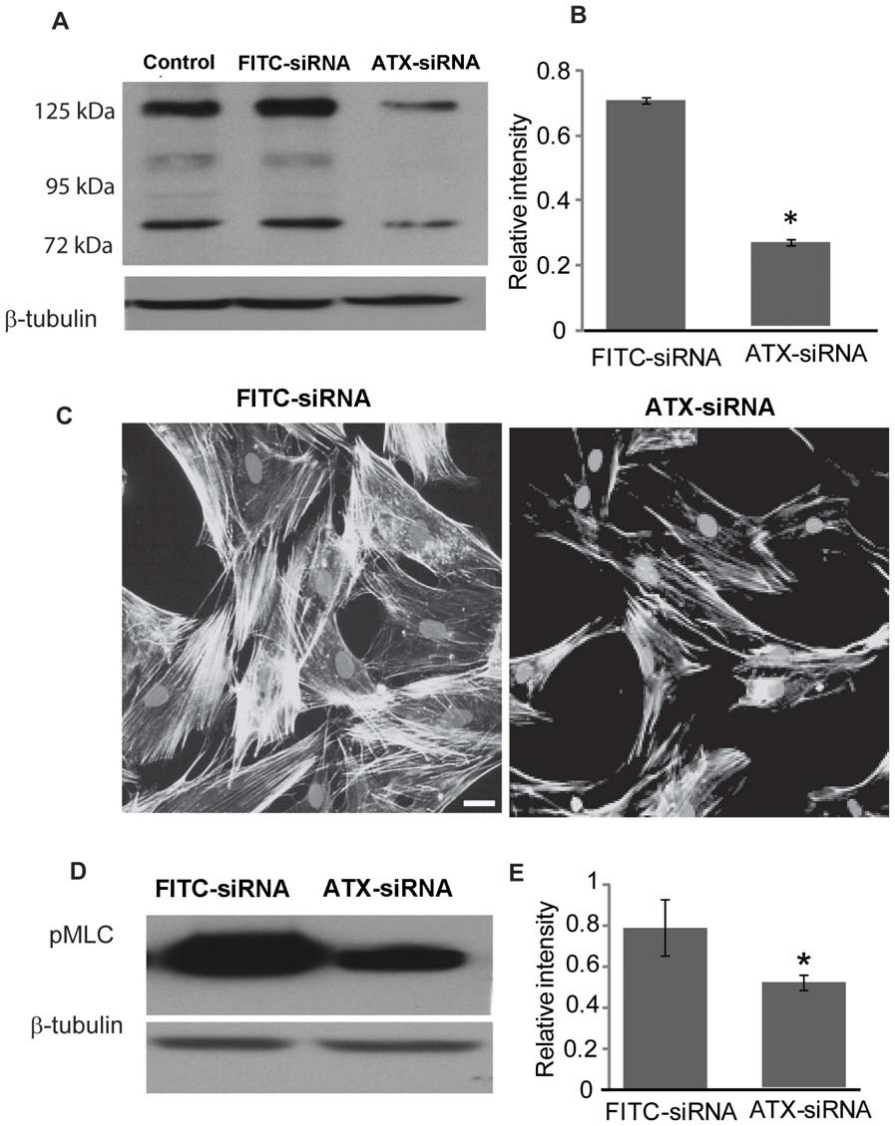
Suppression of ATX expression in human TM cells induces changes in actin cytoskeletal organization and myosin II phosphorylation. A. Primary cultures of human TM cells treated with ATX-specific siRNA revealed significant suppression of ATX expression (different isoforms) relative to cells treated with FITC-conjugated scrambled siRNA control. β-tubulin was used as a loading control. B. Protein levels of one of the variants (∼125 kDa) of ATX were significantly reduced (by >75%) in ATX siRNA treated HTM cells based on densitometric semi-quantification. C. The ATX siRNA treated human TM cells (after 72 hrs) maintained under serum starved conditions for 24 hrs revealed a marked decrease in actin stress fibers (labelled using rhodamine-phalloidin) relative to cells treated with scrambled siRNA (FITC-siRNA). D. ATX siRNA treated human TM cells (for 72 hrs) maintained under serum starved conditions for 24 hrs showed a significant decrease in the levels of phosphorylated myosin light chain (pMLC), a regulatory subunit of myosin II, as compared to cells treated with scrambled siRNA (FITC-siRNA). β-tubulin was blotted as a loading control. E. Changes in the levels of phospho-MLC in the ATX siRNA treated cells compared to the scrambled siRNA treated control, based on densitometric semi-quantitative analysis.

To explore the role of ATX in TM cell morphological and contractile properties, we suppressed the expression of this protein in HTM cells using the small interfering RNA (siRNA) approach. As shown in Figure 8A, treatment of HTM cells with ATX specific siRNA resulted in significant decreases in the intracellular levels of ATX protein relative to cells treated with scrambled FITC-conjugated siRNA and untreated control cells (n = 4). As can be seen from the figure, ATX siRNA treatment affected the levels of different isoforms of ATX. Based on densitometric analysis using β-tubulin as internal standard, the protein levels of a predominant isoforms of ATX (125 kDa) was reduced by 70% in ATX siRNA treated HTM cells (Fig. 8B), while some isoforms (∼100 kDa) were decreased to undetectable levels in HTM cells treated with ATX siRNA (Fig. 8A), relative to control cells treated with scrambled FITC-siRNA. Although the ATX siRNA treated HTM cells did not reveal any obvious changes in cell morphology, F-actin staining by TRITC-phalloidin revealed decreased levels of actin stress fibers compared to the scrambled FITC-siRNA treated controls (Fig. 8C). Additionally, we observed a significant (p<0.03; n = 4) decrease in the levels of phosphorylated myosin light chain, a regulatory subunit of myosin II activity, in HTM cells treated with ATX siRNA, based on immunoblotting analysis (Fig. 8D) and semi-quantitative measurement using densitometric scanning (Fig. 8E).

### Effects of S32826 on cultured TM cells

**Figure 9:**
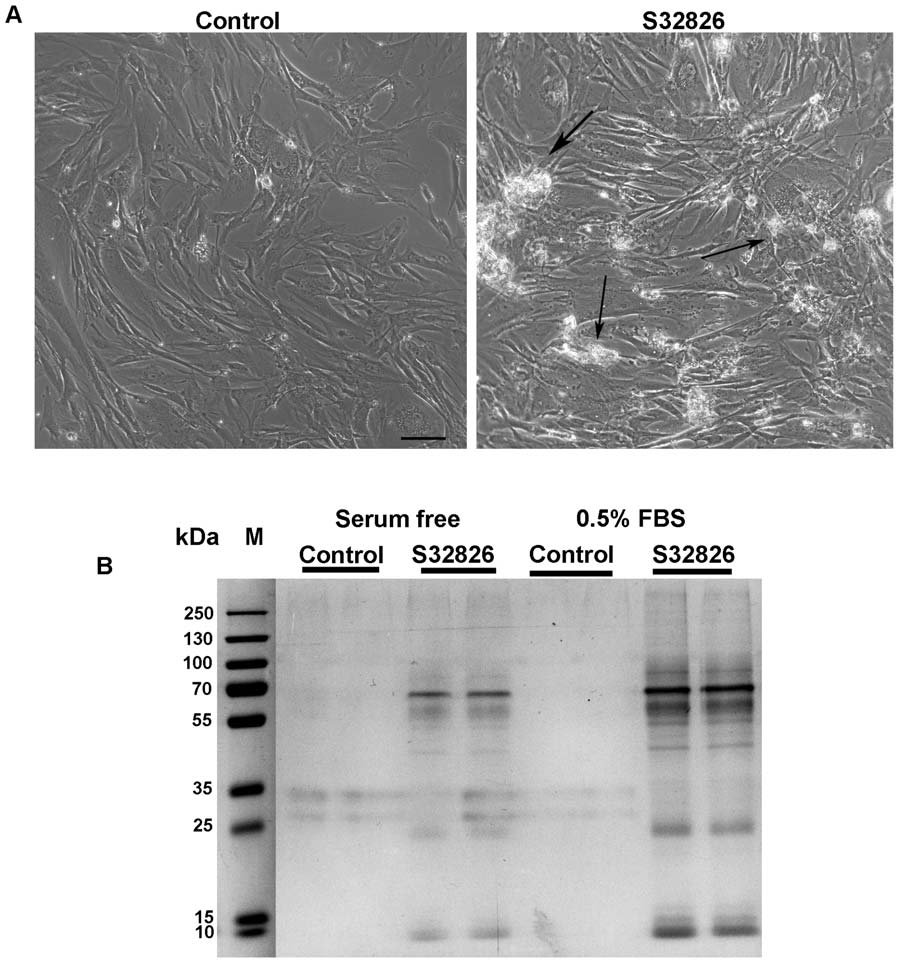
ATX inhibitor-S32826 induced changes in cultured human TM cells. A. Primary cultures of human TM cells cultured either on gelatin-coated glass coverslips or in plastic petridishes were treated with S32826 (10 µM) in the absence of serum or in the presence of 0.5% fetal bovine serum for 24 hrs. Based on phase contrast microscopic examination, treatment of TM cells cultures with drug caused the appearance of membranous floating clumps (arrows) in culture medium, without any noticeable changes in cell morphology and cell adhesion relative to control cells treated with DMSO vehicle. This response appeared to increase progressively with time. B. The membranous floating material generated from drug (S32826) treated cells (A) was extracted from the cell culture media by centrifugation, rinsed with PBS, dissolved in Laemmli sample buffer, separated by SDS-PAGE and stained with gelcode blue as shown in the figure (two independent samples are shown from each treatment). Subsequently, the predominant protein bands were gel extracted and subjected to mass spectrometry-based identification. This analysis revealed presence of proteins involved in lysosomal exocytosis, trafficking and membrane repair as discussed in the results section.

To determine the effects of S32826 on cultured TM cells, confluent cultures of HTM cells (derived from two different donors) maintained under serum free conditions or in the presence of 0.5% fetal bovine serum (FBS) were treated with 5 or 10 µM S32826 for 24 hrs along with respective sham treated controls. Time dependent observations based on phase contrast microscopy revealed presence of large floating membranous clumps in drug-treated cell culture dishes starting as early as 30 minutes from the time of drug addition, and relative to sham treated cells (Fig. 9A. 10 µM drug; arrows). However, drug treatment did not cause changes in cell morphology or cytotoxic response, as assessed by propidium iodide and fluorescein diacetate in live cell labeling (data not shown), [15] or changes in actin stress fiber distribution (data not shown). However, since floating membranous material was consistently noted in drug treated cell cultures in three independent experiments, the material was collected by centrifugation, rinsed with PBS, dissolved in Laemmli sample buffer, and separated by SDS-PAGE. Predominant protein bands were then gel extracted and subjected to mass spectrometry-based identification (Fig. 9B) [30]. Intriguingly, the majority of the proteins found in the floating membranous material shed from drug treated cells belonged to the class of proteins involved in membrane trafficking, exocytosis and membrane repair mechanisms, and included Annexin-2, 5 and 1, myosin-9 and 10, filamin A, radixin, actin, lysozyme C, cathepsin G, α2-macroglobulin, various ECM molecules, heat shock proteins-60, 70 and 90, myoferlin, 14-3-3 adaptor protein zeta/delta, protein S100-09/08, serotransferin, peptidyl-prolyl cis-trans-isomerase, cystatin-A, protein disulfide isomerise and vimentin. The protein profile of membranous material derived from drug treated HTM cells was similar regardless of whether cells had been maintained under serum starved conditions or in medium containing 0.5% FBS.

### Influence of Cyclic Mechanical Stretch on ATX Expression and Secretion in TM cells

**Figure 10:**
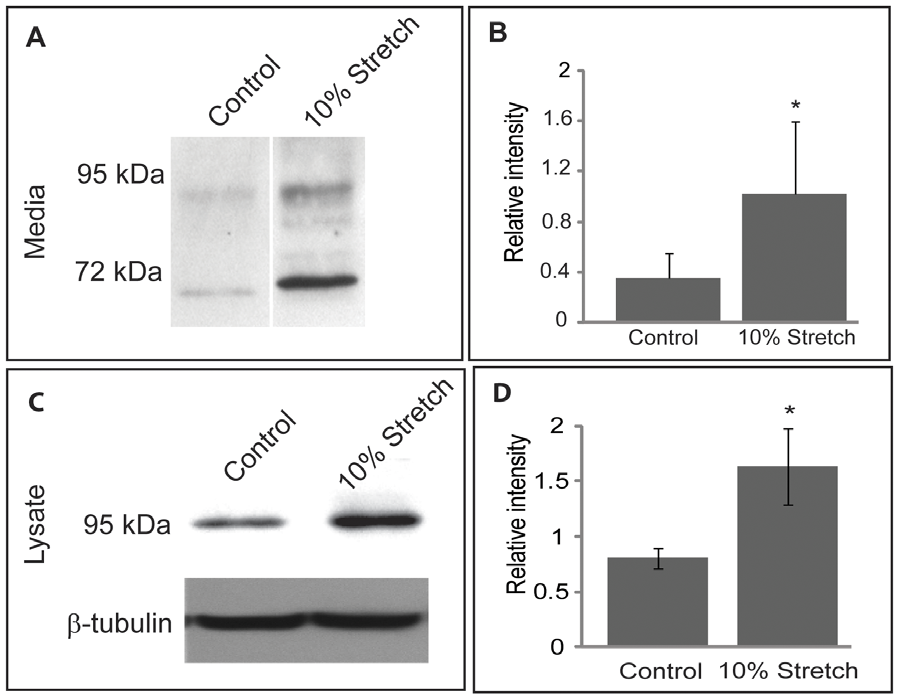
Cyclic mechanical stretch of TM cells induces ATX extracelluar and intracellular protein. A. Confluent cultures of primary porcine TM cells maintained under serum free condition and subjected to 10% cyclical mechanical stretch (one cycle/second) for 17 hrs revealed increased levels of ATX protein levels in the conditioned media as compared to the media derived from the un-stretched control cells based on immunoblot analysis with equal amount (10 µg) of total protein. For a loading control, SDS-PAGE separated proteins were stained with Coomassie blue and the staining intensity was compared (not shown). B. Densitometric semi-quantitative analysis of the immunoblots shown in panel A. *P<0.05, n = 4. C. Cell lysates derived from TM cells subjected to the cyclic mechanical stretch revealed increased levels of ATX protein compared to the samples of unstretched controls based on immunoblot analysis of equal amounts of protein (20 µg). β-tubulin was immunoblotted as a loading control. D. Densitometric semi-quantitative analysis of ATX immunoblots of cell lysates shown in panel C. * P<0.05, n = 4.

TM cells are mechano-sensitive and fluctuations in IOP are thought to influence paracrine and autocrine activity of these cells and thereby influence the homeostatic mechanisms involved in maintenance of normal AH outflow and IOP [8]. To explore the possible involvement of ATX in the effects of cyclic mechanical stretch, serum starved confluent cultures of porcine TM cells (passage 4) were subjected to 17 hrs of cyclic mechanical stretch with one cycle/second with 10% final stretch. In this experiment we used porcine TM cells due to limited availability of HTM cells. Cells exposed to these conditions exhibited significantly elevated levels of ATX both in the conditioned media (Fig. 10A and 10B) and in cell lysates (Fig. 10C and 10D), relative to unstretched control cells as determined by immunoblotting analysis. Figure 10A and 10C show the cyclic stretch-induced changes in ATX protein levels in media and cell lysates, respectively. Densitometric scanning of immunoblots from 4 independent experiments showed a significant (p<0.005) increase in secretory (Fig. 10B) and intracellular (Fig. 10D) ATX protein levels in stretched cells as compared to unstretched control cells.

## Discussion

Increased IOP is a well recognized risk factor for development of POAG, which is a blinding disease [3]. Although lowering IOP is the primary treatment available for different types of glaucoma at present and different classes of ocular hypotensive drugs are used in clinical management of IOP in POAG patients, we lack a mechanism-based and targeted therapy for elevated IOP in glaucoma patients [5], [6]. As part of our ongoing research to identify and characterize molecular mechanisms that lead to ocular hypertension and regulate AH outflow, we have in this study identified ATX/LysoPLD, which is a major source for extracellular LPA, as an abundant protein of human AH and found that AH LysoPLD activity is abnormally elevated in POAG patients. Significantly, topical or intracameral delivery of a small molecular weight chemical inhibitor of ATX (S32826) results in lowering IOP in normal rabbits. Collectively, these observations reveal the importance of the ATX-LPA axis in homeostasis of IOP and its potential as a therapeutic target for development of novel ocular hypotensive agents to treat glaucoma patients. To our knowledge, this is the first report on the possible therapeutic importance of the ATX-LPA axis in treatment of ocular hypertension in glaucoma patients.

The AH contains various extracellular factors of both protein and non-protein makeup which influence trabecular meshwork cell physiology, phagocytic function, contractile responses, autocrine activity and extracellular matrix turnover, several of which have been found to influence AH outflow and IOP [3], [8], [18], [31]. Further, there is convincing experimental and clinical evidence in support of the contention that changes in levels of certain physiologically relevant factors present in the AH might be linked to increased resistance to AH outflow and elevated IOP [11], [18], [19], [32]. To identify such AH factors, we and other investigators have begun to analyze the protein composition of AH derived from the human subjects using recent technical advancements in proteomics capabilities. In our previous studies we demonstrated a role for bioactive lipids (LPA and SIP) and lipid metabolizing enzymes (iPLA2) in regulating AH outflow through the trabecular meshwork [15], [33]. Therefore, we were particularly interested in understanding the role played by AH lipid metabolizing enzymes and exploring the relevance of changes in the levels of these proteins in normal and glaucoma patients.

While it is known that ATX is present in human AH, [34], [35] our quantitative proteomics analysis, revealed that this protein is present in abundant amounts in human AH. ATX, a secretory protein, which was initially characterized as autocrine factor involved in cell motility, proliferation, differentiation and survival, has been identified as an extracellular LysoPLD which generates LPA and to a lesser extent S1P [24], [25], [26], [36], [37], [38]. LPA is a bioactive lipid that in turn regulates several cellular processes including Rho GTPase regulated cell adhesion, contraction and migration by acting through different G-protein coupled receptors [39]. Importantly, abnormal regulation of the ATX-LPA axis has been shown to be associated with various pathological conditions including cancer and fibrosis [24], [37]. Our analysis of AH samples derived from POAG and control (cataract) patients revealed significantly elevated levels of LysoPLD activity (expressed per unit of total protein) in POAG patients.

Although there was a significant difference in racial demography between the glaucoma and control specimens tested in this study, it is unlikely that the 1.8 fold elevation in the LysoPLD activity that we noted in POAG patients relative to age-matched cataract control samples is solely related to differences in the racial demography. Further, there is no known currently available information on racial differences in gene expression profiles of TM or other ocular tissues of the anterior segment of the eye. However, it is important to note that POAG is more prevalent in African Americans and Hispanics relative to Caucasians [40], [41]. While it would have been ideal to determine and confirm whether the observed elevated levels of LysoPLD activity in the AH of POAG patients is associated with elevated levels of LPA, the product of ATX, we could not however, establish this association due to inadequate amounts of AH obtained from each patient.

Based on abundant levels of ATX in AH, and elevated LysoPLD activity in the AH of POAG patients, we hypothesized that inhibition of ATX in the eye would likely impact AH outflow through the trabecular meshwork, and thereby IOP as well. To set the stage for addressing this hypothesis, we first performed *in vitro* studies using a relevant cell type, confirming that TM cells derived from humans and other species express and secrete ATX. Additionally, both secretion and expression of ATX by TM cells was found to be responsive to cyclic mechanical stretch, further supporting the mechanosensitive nature of TM cells and the impact of mechanotransducing stimuli on TM cell physiology [8], [14]. To test whether inhibition of ATX in the AH influences IOP, we evaluated the effects of a well characterized small molecular weight water soluble pharmacological inhibitor of ATX (S32826) [29] on IOP using topical and intracameral drug delivery in rabbits. Both routes of S32826 administration were found to cause significant reductions in IOP in these animals. Since the topical route of administration requires use of millimolar concentrations of S32826, we could not use DMSO to dissolve the drug. Dissolution of drug in sterile filtered water resulted in a solution with an alkaline pH of ∼8–9. Since the drug precipitates out of solution at neutral pH, we used pH adjusted water as sham control in our experiments. S32826 treatment resulted in a dose -dependent ocular hypotensive effect on IOP in rabbits relative to control (sham-treated) eyes. This decrease in IOP was also significant relative to basal IOP in the same animal, and lasted for a minimum of 24 hrs post-treatment in rabbits treated twice a day with 10 mM S32826. It was noted that topical application of alkaline water (sham control) per se caused an upward shift in IOP values by ∼2 to 2.5 mm Hg. The reason for this effect of the sham treatment is not clear. Topical drug application, especially at the 10 mM concentration, was found to cause a transient conjunctival hyperaemia, a response which appears to be associated with drug precipitation on the cornea and conjunctival tissue. However, the decrease in IOP in drug treated eyes was not related to conjunctival hyperaemia since IOP was found to be significantly less in the drug treated animals even after 24 hrs after drug application, by which time there was no evidence of hyperaemia or drug precipitation.

To substantiate the ocular hypotensive effects of S32826 and to circumvent both drug precipitation and resulting conjunctival hyperaemia noted with topical drug delivery in rabbit eyes, S32826 was delivered into the eye anterior chamber by intracameral injections. Importantly, in this case, drug was dissolved initially in DMSO and subsequently diluted in phosphate buffered saline, pH 7.4. Using this method, there was no evidence for drug precipitation or ocular hyperaemia, and IOP was found to be significantly decreased in drug treated eyes by more than 5 mm Hg, with the ocular hypotensive response lasting for more than 48 hrs in response to a single injection of 5 µl of 100 µM S32826. The final concentration of drug in the AH was estimated be in the range of 2 to 2.5 µM based on the volume of AH (approximately 200 to 250 µl) in the adult rabbit eye. Although this experiment provided convincing evidence for the ocular hypotensive effect of S32826, and the ability of S32826 to inhibit LysoPLD activity in rabbit AH was confirmed using an *in vitro* assay, we could not validate that the ocular hypotensive response of S32826 was indeed associated with decreased levels of LPA. Our preliminary attempts to determine levels of LPA in the limited amounts of AH obtained from rabbit eyes by mass spectrometric analysis were not successful. Further work is needed to establish protocols for determining LPA levels in AH. However, S32826 has been shown to decrease LPA levels produced by adipocytes [29].

Finally, siRNA-mediated suppression of ATX expression in TM cells resulted in a decrease in actin stress fibers and myosin light chain phosphorylation, indicating a role for ATX in cell contractile properties, cell tension and cell adhesive properties of TM cells. Consistent with these observations, our previous studies demonstrated that LPA, which is a direct product of ATX activity, induces formation of actin stress fibers, focal adhesions and myosin light chain phosphorylation and Rho GTPase activation in TM cells [15]. Further, in contrast to the ocular hypotensive effects of the ATX inhibitor S32826 noted in this study, perfusion of LPA has been demonstrated to decrease AH outflow facility, leading to increased IOP [15]. Intriguingly, S32826 showed no detectable effects either on cell morphology or actin cytoskeletal reorganization of TM cells maintained in serum free media. Instead, drug treated TM cell cultures maintained either under serum-starved conditions or with 0.5%FBS were observed to generate cell-derived membranous floating material with no evidence of a cytotoxic response. The analysis of protein composition of this material by mass spectrometry analysis confirmed presence of proteins involved in membrane trafficking, repair and exocytosis. It is not clear whether inhibition of autotaxin, which is known to act locally at cell surface by interacting with integrins and to produce LPA close to the cell surface receptors, influences membrane recycling properties in TM cells [42], [43], [44].

In conclusion, this initial study reports the ocular hypotensive effect of an ATX inhibitor in live rabbits, suggesting potential significance of ATX as a therapeutic target for lowering IOP in glaucoma patients. Further studies are necessary to delineate the molecular mechanisms by which inhibition of ATX mediates decrease of IOP.

## Methods

### Ethics Statement

#### Animals

This study was carried out in strict accordance with the recommendations in the Guide for the Care and Use of Laboratory Animals of the National Institutes of Health and Association for Research in Vision and Ophthalmology. The protocol was approved by the Committee on the Ethics of Animal Experiments of the Duke University School of Medicine (Protocol Number: A094-10-04). Intracameral injections were performed under ketamine/xylazine anesthesia, and all efforts were made to minimize pain and suffering during experiments.

#### Human Subjects

All research involving collection of human aqueous humor samples has been approved by the Duke University School of Medicine's institutional review board (IRB). Informed written patient consent was obtained prior to sample collection and the principles expressed in the Declaration of Helsinki were followed. This study does not involve minors or children.

### Reagents

RNeasy Mini kit (Qiagen, Valencia, CA), Advantage RT for PCR kit and Advantage cDNA PCR kit (BD Biosciences Clontech, Palo Alto, CA) were procured from the respective commercial sources. Mouse monoclonal anti-tubulin antibodies and Tetramethyl rhodamine isothiocyanate (TRITC)–conjugated phalloidin were from Sigma-Aldrich (St. Louis, MO). Polyclonal phospho-MLC antibody (C.No. 36745) was from Cell Signalling Technology (Danvers, MA). L-α-Lysophosphatidylcholine (C.No. 830071P) was from Avanti Polar Lipids, Inc. (Alabaster, AL). ATX polyclonal antibody (C. No. 10005375) and its blocking peptide (C. No. 10007193) were obtained from Cayman Chemicals (Ann Arbor, MI), and S32826 (ATX inhibitor) were from Cayman Chemicals (C. No. 13664) and Sigma-Aldrich (C. No. S1825). Amplex Red PLD Assay Kit (C.No. A12219) was from Molecular Probes/Invitrogen (Grand Island, NY). Fetal Bovine Serum (FBS) and Dulbecco's Modified Eagle's Medium (DMEM) with low glucose were obtained from Gibco-BRL (Gaithersburg, MD). Bio-Rad protein assay reagent (C. No. 500-0006) was purchased from Bio-Rad. FITC (Fluorescein isothiocyanate) conjugated scrambled siRNA (C. No. sc-36869) and human ATX siRNA (C. No. sc-44906) were from Santa Cruz Biotechnology (Santa Cruz, CA).

### Collection of Aqueous Humor

#### Humans

Aqueous humor was collected from patients undergoing elective cataract surgery or surgery for glaucoma at the Duke Eye Center by the glaucoma and comprehensive clinical service clinicians. A cannula attached to a tuberculin syringe was inserted into the anterior chamber through a paracentesis tract at the start of surgery and AH was slowly aspirated until shallowing of the anterior chamber. The sample was transferred from the syringe to a microcentrifuge tube and was centrifuged at 3000 rpm for 10 min at 4°C. The supernatant was collected and stored at −80°C until further use.

#### Animals

Mouse AH was collected from 1 year old male C57BL/6 mice. The mice were first sacrificed using sodium phenobarbital intraperitoneally and a 25 µl Hamilton syringe (22 s/2″/2) was carefully inserted into the anterior chamber of the eye to withdraw the AH. Porcine eye AH was collected using a sterile insulin syringe (29G) from freshly enucleated eyes obtained from a local slaughterhouse. AH from anaesthetized (using ketamine/xylazine) New Zealand white rabbits was collected using an insulin syringe (29G). AH derived from these different species was centrifuged at 3000 rpm, 4°C for 10 minutes and the supernatant was collected and stored at −80°C till further use.

### Quantitative Proteomics of human AH

Briefly, human AH samples were collected from 8 patients during cataract surgery, centrifuged at 3000 RPM, 4°C for 10 minutes and equal volumes of supernatant from individual specimens were pooled. Protein content of the pooled specimen was determined using a Bio-Rad dye protein assay reagent with bovine serum albumin as the standard. The AH sample was reduced with 10 mM dithiothreitol, alkylated with 20 mM iodoacetamide and digested with trypsin in the presence of 0.1% w/v RapiGest SF surfactant (Waters). The sample was spiked with 50 fmol of yeast alcohol dehydrogenase digest (Waters Mass Prep) per µg of total AH protein. One µg of protein from the digested AH was injected onto a nanoAcquity LC system, coupled to a Synapt HDMS mass spectrometer. The sample was analyzed in triplicate using MS^E^ (data-independent acquisition) for quantitative/qualitative analysis, followed by a single data-dependent analysis (DDA) for supplementary identifications. Data were searched against the Swissprot Human (v57.5) database, with 20 ppm precursor and 0.04 Da product ion mass accuracy and trypsin specificity, with either PLGS 2.4/IdentityE (MSE) or Mascot v2.2 (DDA) search engine. Data was loaded into Scaffold 2 Proteome Software version 2.06 to generate a list of the most abundant proteins detected by mass spectrometry. Raw data alignment, feature detection, and label-free quantitation were performed within Rosetta Elucidator v3.3 software. The fmol quantity of each protein (per µg injected) was performed according to the method of Silva et al. [45] for the 89 proteins detected based on 2 or more peptides using yeast alcohol dehydrogenase as an internal standard. These analyses were performed at the Duke Proteomics Core Facility.

### TM cell cultures

Human primary TM cells were cultured from fresh corneal rings donated by the Duke Ophthalmology Clinical Service after they were used for corneal transplantation as described previously [22]. Porcine primary TM cells were isolated and cultured using collagen IV digestion as described earlier by us [46]. Cell passages between 4 and 6 were used in this study.

### LysoPLD assay

LysoPLD enzyme activity was assayed by measuring hydrolysis of lysophosphatidylcholine to choline and lysophosphatidic acid as described by Gijsbers et al. [27]. Briefly, choline derived from the hydrolysis of lysophosphatidylcholine was coupled with choline oxidase to oxidize choline into betaine and H_2_O_2_. Subsequently, the H_2_O_2_ generated reacts with Amplex Red reagent in the presence of horseradish peroxidase to generate resorufin, which is measured using a fluorescence spectrometer with excitation and emission filters at 530 nm and 590 nm, respectively. The original PLD assay was modified for lysoPLD by replacing the substrate phosphatidylcholine with lysophosphatidylcholine. The enzyme reactions were performed in duplicate in a final volume of 200 µl at 37°C for 30 minutes using a 96 well microtiter plates and Molecular Probe Spectramax M3. H_2_O_2_ standards, heat inactivated AH control and enzyme assays lacking lysophosphatidylcholine substrate were run simultaneously. Enzyme activity was expressed as µM H_2_O_2_ generated per mg of total AH protein.

### Immunofluorescence

TM cells were plated on glass coverslips coated with 2% gelatin and grown till they were 80% confluent. After treatments in serum-free media, they were washed twice with 1× phosphate buffered saline (PBS) and fixed in 4% paraformaldehyde for 15 min at room temperature. Cells were permeabilized in 0.5% Triton-X 100 for 15 minutes, and blocked in serum buffer containing 10% FBS. Following this, cells were incubated with respective primary and Alexa fluor-conjugated secondary antibodies as we described earlier [22]. Finally, coverslips were washed and mounted onto glass slides with Aqua Mount (Lerner Laboratories, Pittsburg, PA) and observed under a Nikon confocal system (C1 Digital Eclipse).

### Immunoblotting

Total cell lysates were prepared from confluent serum-starved TM cells. Briefly, cells were washed twice with cold PBS, and collected into lysis buffer containing 10 mM Tris, pH 7.4, 0.2 mM MgCl_2_, 5 mM N-ethylmaleimide, 0.4 mM iodoacetamide and 60 µM PMSF and protease inhibitor (cocktail tablets complete, Mini, EDTA-free, Roche, Basel, Switzerland), and phosphatase inhibitors (Phosphostop, Basel, Switzerland), sonicated on ice (probe sonicator, followed by bath sonicator for 30 min) and centrifuged at 800 g, 4°C for 15 min. The supernatant was collected and protein was measured as described below. Media was collected and centrifuged at 3000 rpm for 10 min at 4°C to remove cellular debris. The supernatant was concentrated using Millipore Centrifugal Filter Units with <30 kDa cutoff. The filters were centrifuged at 2500 rpm at 4°C. Bio-Rad dye protein assay reagent was used to estimate the protein concentrations in both cell lysate and media samples using the Bradford method. Equal amounts of protein were dissolved in Laemmli buffer, boiled for 5 minutes, and separated by SDS-PAGE using 8% acrylamide gels. Proteins were transferred to nitrocellulose membrane, which were then incubated with LysoPLD polyclonal antibody (diluted 1∶250 in TBST) overnight at 4°C. Immunoblots were developed using appropriate HRP (horse radish peroxidase)-conjugated secondary antibody and enhanced chemiluminescence, as we described earlier [22]. Densitometry of scanned films was performed using Adobe Photoshop CS3 Software, and results expressed relative to the loading control (β-tubulin).

### Myosin Light Chain (MLC) Phosphorylation

Changes in TM cell myosin light chain phosphorylation status were determined by immunoblot analysis using a phospho-MLC specific antibody and urea-glycerol gel electrophoresis as we described earlier [15]. Densitometry of scanned films was performed using Adobe Photoshop CS3 Software, and results expressed relative to the loading control (β-tubulin).

### RT-PCR

Total RNA was extracted using RNeasy Micro kit (Qiagen, Valencia, CA) per the user manual from P4 confluent cultures of HTM cells derived from human donors aged 33, 54 and 75 years. 2 µg of total RNA was used for first-strand synthesis using Superscript first-strand synthesis system for RT-PCR (Life Technologies, Carlsbad, CA). The resultant cDNA was used to amplify DNA specific to ATX isoforms in a total volume of 50 µl with 1.5 mM MgCl_2_, 0.2 mM dNTPs, 2 U *Taq* polymerase (Life Technologies), and 0.5 µM sense and antisense primers for the α, β, and γ variants of ATX. GAPDH primers were used as positive control. The primer sequences (forward/reverse) used to amplify the different isoforms of ATX are listed below:

1. α – ATGGATTACAGCCACCAAGC/TCTTCCATTCCATGGTCTCC
2. β- TCGGCCCTGAGATGACAAATC/GAGGTGTCTCTCTTCTGTAG
3. γ – ATGTCCGTGTTTCTCCGAGT/ATTGCAGCTCTCCTCGTTGT
4. GAPDH – TGCACCACCAACTGCTTAGC/GGCATGGACTGTGGTCATGAG.

The amplification was carried out using GeneAmp Amplification System (Applied Biosciences) with a denaturation step at 94°C for 4 min, followed by 94°C for 1 min, annealing at 57°C for 60 s, and extension at 72°C for 60 s. This cycle was repeated 30 times with a final step at 72°C for 7 min. The resulting products were separated by 1% agarose gel electrophoresis and visualized by ethidium bromide staining and using a Fotodyne Trans-illuminator (Fotodyne Inc., Wisconsin). Control reactions containing no reverse transcriptase were run simultaneously.

### Cyclic Mechanical Stretch

Porcine TM cells between passages 4 and 5 were plated onto BioFlex six-well plates pre-coated with collagen (Flexcell International, Hillsborough, NC). Once they reached confluence, cells were serum starved for 24 hrs and then stretched using the FlexCell FX-4000 system (Flexcell International, Hillsborough, NC). A cyclic stretch of 1 cycle/second was applied to the experimental plates for 17 hrs at 37°C as we described earlier [47]. Control plates were incorporated into the same system, but did not undergo stretch. Experimental plates were exposed to a total stretch of 10% as compared to control plates. After stretch treatment, media were collected, centrifuged (3000 rpm, 4°C for 10 minutes) and supernatants were used for detection of ATX by immunoblotting. The cells were scraped and lysates were prepared for Western blotting analysis as previously described.

### siRNA Treatment

HTM cells (passaged between 3 to 5 cycles; derived from donors aged 16, 18 and 74 years) were cultured in 100 cm plates in DMEM with 10% FBS, until they reached about 80% confluency. Medium (containing 10% FBS) was changed a day before and on the day of transfection. Cells were trypsinized and counted prior to transfection, and 5×10^5^ cells were used for each nucleofector transfection. Two different controls: untransfected control, and FITC-conjugated scrambled siRNA control were set up for these experiments. The Amaxa Nucleofector (program T023) and Basic Endothelial Cell Nucleofector Kit (C. No. VP1-1001) were used and the kit specifications were strictly adhered to. Post transfection, cells were plated in 6-well plates and coverslips coated with 2% gelatin for immunostaining. Cells were changed into fresh medium on the day following transfection. FITC fluorescence was detected after 24–36 hours of transfection. Prior to cell lysate preparation for Western blot analysis, cells were changed into serum-free media overnight and the standard procedure was followed for cell lysate preparation. Coverslips were fixed for actin filament staining using TRITC-phalloidin as described earlier.

### Animal experimentation

#### Topical drug effects

In the first set of experiments, we used four Dutch-Belted adult male rabbits. One eye was treated with drug while the other (contralateral) eye was used as control (sham treatment). Basal IOPs were measured using a pneumotonometer (Model 30, Medtronic) for a week before beginning the experiments to monitor for any irregularities in IOP. The ATX inhibitor (S32826; Sigma) was dissolved in sterile autoclave distilled water (resulting in an alkaline pH∼8) to obtain a desired concentration (2–10 mM) for the topical dosing. pH adjusted (pH∼8) distilled water served as a control for the contralateral eye. Before administering drug or sham control vehicle, the eyes were treated with a drop of topical anesthetic (0.5% proparacaine HCl). The animals were awake during the IOP measurements. Basal IOPs were measured prior to drug treatments. 35 µl of the drug or sham control vehicle were applied to the respective eyes. This volume was found to be appropriate, covered the entire cornea and did not spill over from the eye. During drug application, the rabbit eyes were kept open physically to prevent blinking for a minute before applying a second aliquot of 35 µl drug solution. In the first set of rabbits, 3 different concentrations of the drug (10, 2 and 5 mM) were tested in separate experiments (sequentially). Only for the 10 mM concentration, a second dose of drug was administered 5–6 hrs (typically at ∼4 PM) after the first dose (typically at ∼10 AM). The effects of 10 mM drug were repeated in a second set of rabbits under identical conditions as the first experiment, using 5 additional rabbits. IOPs were measured at 2, 5, 7, 24, 32 and 50 hrs after the first dose of drug application. An average of four readings was used to plot the IOP data for each time point. For the lower concentrations of drug (5 mM and 2 mM), only one morning dose was applied every day (∼10/11 AM) for 5 days, and IOPs were monitored twice a day. For the concentration response experiments, animals received sufficient recovery time after each concentration experiment was completed, and the next concentration experiment was tested only after IOP returned to the basal or normal level. The rabbits from the first set of experiments were sacrificed after they received the 5 mM drug for 5 days, after which AH was collected and enucleated eyes fixed for histological analysis.

#### Intracameral drug effects

Due to drug precipitation on the corneal surface and conjunctival hyperaemia associated with topical drug delivery, an intracameral injection experiment was designed using a new batch of four rabbits (same strain as the previous experiments). The volume of AH in adult rabbits was estimated to be ∼200–250 µl based on our previous observations. For this study, the drug stock was prepared initially in DMSO and subsequently diluted in PBS, such that the final DMSO concentration in the aqueous humour would be ∼0.05%. Prior to intracameral injections, basal IOPs were measured in awake rabbits as described above. The rabbits were anesthetized using intramuscular injections of ketamine and xylazine cocktail, followed by a topical drop of 0.5% proparacaine HCl. An operating microscope was positioned over the eye undergoing surgery and the anterior chamber was entered in the superotemporal quadrant using a 0.3 cc insulin syringe positioned at an angle and 5 µl of drug (100 µM stock in PBS with 2% DMSO) was injected into the eye anterior chamber. The injections and follow-up ocular changes were performed by a board certified glaucoma specialist. The contralateral eye served as the non-injected control. IOP values in both eyes were measured at 24, 48, 72, 96, and 120 hrs post drug injections, until the pressures in the injected eyes returned to normal. During this time, the eyes were monitored for ocular changes using a slit lamp. Once these rabbits recovered from drug injections after 2 weeks, the contralateral eye was injected with 5 µl of PBS containing 2% DMSO and served as the DMSO-injected sham control. IOPs were measured in both eyes at 24, 48, 72, 96, and 120 hrs post injection.

### Histology

Rabbit eyes from the topical drug experiment (5 mM drug) were enucleated, rinsed in 1× PBS and fixed in 4% paraformaldehyde (PFA) for 1 hr at room temperature. After this, the lens was carefully removed and the anterior segment was cut into two halves, one half being processed for assessment of corneal toxicity. Briefly, tissue was immersed in 10% buffered formalin at room temperature and sent for pathological analysis, where it was processed and stained with haematoxylin and eosin (H&E). The other half was processed for histological changes at the AH outflow angle. The tissue was immersed in 2.5% glutaraldehyde, 50 mM cacodylate buffer (pH 7.2) containing 4% sucrose and 2 mM CaCl_2_ for 2 hrs, then transferred to 10% buffered formalin for 48 hrs. The specimens were then embedded in methyl methacrylate, and 2 µm sections were cut and stained with hematoxylin and eosin stain using conventional methods. Micrographs were captured using a Zeiss light microscope.

### Statistical analysis

In figures, all data are presented as mean ± SEM. The Wilcoxon Rank Sum test of differences in medians was performed to compare the ATX enzyme activity between glaucoma and cataract controls with one-way Chi-square approximation. IOP changes between drug treated and sham treated rabbits were assessed for significance using the Wilcoxon Rank Sum test. IOP changes within the same group before and after drug treatment was tested using the paired t-test. Racial difference between glaucoma and cataract groups was tested by the Fisher's exact test. Student's t-test was used for evaluating significance in biochemical studies performed with cultured cells. A p-value<0.05 was considered significant. A box-and-whisker plot was used to represent the AH LysoPLD activity in glaucoma and cataract control subjects.

## Supporting Information

Table S1 **Quantitative proteome of aqueous humor from control (cataract) eyes with proteins listed in order of abundance.** Values represent mean ± standard deviation (SD).
(DOCX)
