# Supplementary material for: Autotaxin-Lysophosphatidic Acid Axis Is a Novel Molecular Target for Lowering Intraocular Pressure
Source: PLoS One. 2012 Aug 20;7(8):e42627. doi: 10.1371/journal.pone.0042627 (PMC3423407; doi:10.1371/journal.pone.0042627)
Supplement: Table S1 — Quantitative proteome of aqueous humor from control (cataract) eyes with proteins listed in order of abundance. Values represent mean ± standard deviation (SD). (DOCX) [file pone.0042627.s001.docx]

**Table S1.**

| **Swiss-Prot Number** | **Protein Common Name** | **fmol/μg AH Protein** |
| --- | --- | --- |
| **P02768** | Serum albumin | 2059.9 ± 22.6 |
| **P02787** | Serotransferrin | 914.1 ± 26.3 |
| **P01834** | Ig kappa chain C region | 675.0 ± 7.0 |
| **P01857** | Ig gamma-1 chain C region | 573.7 ± 14.6 |
| **P41222** | Prostaglandin-H2 D-isomerase | 481.7 ± 5.6 |
| **P10909** | Clusterin | 456.7 ± 10.6 |
| **P01861** | Ig gamma-4 chain C region | 366.8 ± 4.6 |
| **P01859** | Ig gamma-2 chain C region | 343.5 ± 2.6 |
| **P0CG04** | Ig lambda chain C regions | 338.8 ± 13.4 |
| **P02765** | Alpha-2-HS-glycoprotein | 328.2 ± 4.4 |
| **P36955** | Pigment epithelium-derived factor | 323.4 ± 8.1 |
| **P02790** | Hemopexin | 316.7 ± 10.3 |
| **P02763** | Alpha-1-acid glycoprotein | 222.4 ± 2.1 |
| **P01009** | Alpha-1-antitrypsin | 208.0 ± 4.7 |
| **Q9NWS1** | UPF0419 protein C12orf48 | 192.3 ± 4.8 |
| **P01034** | Cystatin-C | 160.1 ± 3.2 |
| **P02774** | Vitamin D-binding protein | 156.2 ± 2.0 |
| **P01008** | Antithrombin-III | 148.7 ± 1.2 |
| **P01011** | Alpha-1-antichymotrypsin | 136.0 ± 3.9 |
| **Q13822** | Autotaxin (ENPP2) | 133.4 ± 2.2 |
| P01620 | Ig kappa chain V-III region SIE | 105.8 ± 4.7 |
| **P22352** | Glutathione peroxidase 3 | 96.0 ± 2.1 |
| **P00738** | Haptoglobin | 91.0 ± 1.9 |
| **P00450** | Ceruloplasmin | 86.5 ± 1.3 |
| **P04264** | Keratin, type II cytoskeletal 1 | 85.3 ± 0.3 |
| **P19652** | Alpha-1-acid glycoprotein 2 | 80.6 ± 1.5 |
| **P05155** | Plasma protease C1 inhibitor | 78.3 ± 2.4 |
| **P01024** | Complement C3 | 72.0 ± 1.5 |
| **P06396** | Gelsolin | 71.2 ± 2.7 |
| **P18135** | Ig kappa chain V-III region HAH | 70.8 ± 2.3 |
| **P02749** | Beta-2-glycoprotein 1 | 67.0 ± 0.2 |
| **P01876** | Ig alpha-1 chain C region | 66.2 ± 2.5 |
| **P02766** | Transthyretin | 64.9 ± 1.2 |
| **P10745** | Retinol-binding protein 3 | 61.2 ± 1.1 |
| **P02538** | Keratin, type II cytoskeletal 6A | 60.0 ± 3.1 |
| **P61626** | Lysozyme C | 58.6 ± 7.0 |
| **P61769** | Beta-2-microglobulin | 57.9 ± 1.3 |
| **P0C0L5** | Complement C4-B | 55.8 ± 1.8 |
| **P13645** | Keratin, type I cytoskeletal 10 | 53.9 ± 4.3 |
| **P00330** | Alcohol dehydrogenase 1 (internal standard) | 50.0 ± 2.1 |
| **P08779** | Keratin, type I cytoskeletal 16 | 49.3 ± 0.7 |
| **P01023** | Alpha-2-macroglobulin | 48.4 ± 1.6 |
| **P02647** | Apolipoprotein A-I | 46.7 ± 2.4 |
| **P04217** | Alpha-1B-glycoprotein | 42.6 ± 1.2 |
| **Q9UBP4** | Dickkopf-related protein 3 | 39.6 ± 0.6 |
| **P25311** | Zinc-alpha-2-glycoprotein | 37.2 ± 0.5 |
| **P01860** | Ig gamma-3 chain C region | 33.7 ± 0.9 |
| **P36222** | Chitinase-3-like protein 1 | 33.3 ± 0.4 |
| **P04196** | Histidine-rich glycoprotein | 31.7 ± 1.0 |
| **P00751** | Complement factor B | 31.6 ± 0.2 |
| **P01766** | Ig heavy chain V-III region BRO | 27.0 ± 0.9 |
| **P02533** | Keratin, type I cytoskeletal 14 | 26.7 ± 0.2 |
| **P35527** | Keratin, type I cytoskeletal 9 | 25.0 ± 0.6 |
| **O94985** | Calsyntenin-1 | 25.0 ± 0.4 |
| **Q12805** | EGF-containing fibulin-like extracellular matrix protein 1 | 23.2 ± 0.2 |
| **P01765** | Ig heavy chain V-III region TIL | 22.9 ± 0.9 |
| **P35908** | Keratin, type II cytoskeletal 2 epidermal | 22.4 ± 0.5 |
| **P35908** | Osteopontin | 21.8 ± 1.3 |
| **P01019** | Angiotensinogen | 21.0 ± 1.1 |
| **P07339** | Cathepsin D | 19.5 ± 0.6 |
| **P22914** | Beta-crystallin S | 18.3 ± 0.9 |
| **Q9UBM4** | Opticin | 17.1 ± 0.4 |
| **P61916** | Epididymal secretory protein E1 | 16.6 ± 0.4 |
| **P01042** | Kininogen-1 | 16.4 ± 0.8 |
| **P04433** | Ig kappa chain V-III region VG (Fragment) | 15.9 ± 0.2 |
| **P01625** | Ig kappa chain V-IV region Len | 15.3 ± 0.8 |
| **P00747** | Plasminogen | 14.5 ± 0.1 |
| **P02753** | Retinol-binding protein 4 | 14.4 ± 0.1 |
| **Q16270** | Insulin-like growth factor-binding protein 7 | 13.3 ± 0.3 |
| **P01600** | Ig kappa chain V-I region Hau | 13.1 ± 0.5 |
| **P02760** | Protein AMBP | 12.8 ± 0.2 |
| **P04259** | Keratin, type II cytoskeletal 6B | 11.9 ± 0.1 |
| **P02750** | Leucine-rich alpha-2-glycoprotein | 10.9 ± 0.7 |
| **P01781** | Ig heavy chain V-III region GAL | 10.7 ± 0.1 |
| **P02649** | Apolipoprotein E | 9.3 ± 0.5 |
| **Q9UPQ4** | Tripartite motif-containing protein 35 | 9.0 ± 1.0 |
| **P01877** | Ig alpha-2 chain C region | 8.8 ± 0.2 |
| **P68871** | Hemoglobin subunit beta | 7.2 ± 0.4 |
| **P01743** | Ig heavy chain V-I region HG3 | 6.6 ± 0.2 |
| **P04004** | Vitronectin | 6.4 ± 0.1 |
| **P69905** | Hemoglobin subunit alpha | 6.0 ± 0.3 |
| **P13647** | Keratin, type II cytoskeletal 5 | 4.5 ± 0.3 |
| **P06727** | Apolipoprotein A-IV | 4.0 ± 0.2 |
| **P16870** | Carboxypeptidase E | 2.7 ± 0.3 |
| **P08697** | Alpha-2-antiplasmin | 2.3 ± 0.2 |
| **P04206** | Ig kappa chain V-III region GOL | 1.9 ± 0.3 |
| **P05090** | Apolipoprotein D | 1.6 ± 0.1 |
| **P02671** | Fibrinogen alpha chain | 0.9 ± 0.1 |
| **P02675** | Fibrinogen beta chain | 0.9 ± 0.1 |
| **P04908** | Histone H2A type 1-B/E | 0.5 ± 0.0 |
